# Lightweight MRI Brain Tumor Segmentation Enhanced by Hierarchical Feature Fusion

**DOI:** 10.3390/tomography10100116

**Published:** 2024-10-01

**Authors:** Lei Zhang, Rong Zhang, Zhongjie Zhu, Pei Li, Yongqiang Bai, Ming Wang

**Affiliations:** 1 Ningbo Industrial Vision and Industrial Intelligence Lab, Zhejiang Wanli University, Ningbo 315100, China; zhlangl01@hotmail.com (L.Z.); 2022883025@zwu.edu.cn (R.Z.); 2022882023@zwu.edu.cn (P.L.); yongqiangbai@zwu.edu.cn (Y.B.); 2 Faculty of Information Science and Engineering, Ocean University of China, Qingdao 266100, China; acwarming@stu.ouc.edu.cn

**Keywords:** MRI brain tumor segmentation, lightweight, hierarchical feature fusion, macro perception, micro focus

## Abstract

Background: Existing methods for MRI brain tumor segmentation often suffer from excessive model parameters and suboptimal performance in delineating tumor boundaries. Methods: For this issue, a lightweight MRI brain tumor segmentation method, enhanced by hierarchical feature fusion (EHFF), is proposed. This method reduces model parameters while improving segmentation performance by integrating hierarchical features. Initially, a fine-grained feature adjustment network is crafted and guided by global contextual information, leading to the establishment of an adaptive feature learning (AFL) module. This module captures the global features of MRI brain tumor images through macro perception and micro focus, adjusting spatial granularity to enhance feature details and reduce computational complexity. Subsequently, a hierarchical feature weighting (HFW) module is constructed. This module extracts multi-scale refined features through multi-level weighting, enhancing the detailed features of spatial positions and alleviating the lack of attention to local position details in macro perception. Finally, a hierarchical feature retention (HFR) module is designed as a supplementary decoder. This module retains, up-samples, and fuses feature maps from each layer, thereby achieving better detail preservation and reconstruction. Results: Experimental results on the BraTS 2021 dataset demonstrate that the proposed method surpasses existing methods. Dice similarity coefficients (DSC) for the three semantic categories ET, TC, and WT are 88.57%, 91.53%, and 93.09%, respectively.

## 1. Introduction

Brain tumors, posing a significant threat to human health, represent a severe neurological disorder [1,2]. Gliomas, the most prevalent type of brain tumor, are classified into low-grade gliomas (LGG, grades 1–2) and high-grade gliomas (HGG, grades 3–4). HGGs, characterized by rapid infiltration into adjacent tissues, are malignant gliomas that present formidable treatment challenges and lead to reduced survival rates. Early detection, localization, and segmentation of tumors at the LGG stage via medical imaging can facilitate timely surgical interventions and improve patient outcomes. Brain tumor examination and analysis predominantly rely on Magnetic Resonance Imaging (MRI). However, challenges, including complex tumor shapes and blurred edges, persist [3,4]. Thus, a precise MRI brain tumor segmentation method is urgently needed to aid clinical diagnosis and treatment.

Brain tumor segmentation methods primarily encompass two categories: 2D and 3D segmentation methods based on deep learning. Two-dimensional methods dissect MRI images into two-dimensional images, which are then processed using 2D CNNs to extract features and classify pixels. Zhao et al. [5] pioneered the use of CNNS to design an automatic brain tumor segmentation model for 2D sliced MRI images. However, this approach neglected local detail information and contextual information about the brain tumor. To capture more contextual information, Dong et al. [6] pioneered the application of the U-Net [7] in medical image segmentation. The simple architecture of the U-Net enables the network to consider contextual information. However, the feature extraction capability of the U-Net could be stronger, prompting researchers [8,9] to refine the U-Net. Kong et al. [8] incorporated a feature pyramid module into the U-Net to enhance features by capturing multi-scale semantic information. Chen et al. [9] adjusted the convolution kernel size in each layer of the U-Net encoder to extract features with different receptive fields. Despite the advancement of 2D methods for brain tumors, issues like minor changes between adjacent slices remain. While 2D CNNs process complex brain tumor image slices, they overlook the three-dimensional correlation and often disrupt potential cross-slice continuity, leading to a loss of contextual information.

In contrast, 3D segmentation methods effectively address the abovementioned issues by considering multiple slices simultaneously. They allow for a more comprehensive capture of the three-dimensional shapes and locations of the brain tumors, better meeting the needs of biomedical applications. Inspired by the U-Net, Cicek et al. [10] constructed a 3D U-Net model for volumetric data, converting the input from two-dimensional to three-dimensional images and significantly improving performance. However, this method still could not overcome the inherent essence of the U-Net, namely weak feature extraction capability. To address this problem, Kamnitsas et al. [11] proposed a dual-pathway 3D CNN segmentation network. Its ability to simultaneously process features at multiple scales enhanced its representation capability. However, it did not pay much attention to feature residuals, preventing the network from training deeper and causing information loss. To this end, Alom et al. [12] proposed the R2U-Net based on a recurrent residual network. The network allows the model to train deeper, while the recurrent network makes feature extraction more comprehensive. It achieved better performance, but the recurrent mechanism substantially increased the complexity, necessitating higher hardware requirements. Unlike the recurrent mechanism, Jiang et al. [13] designed a two-stage 3D U-Net model. The first stage performs rough segmentation through a shallow network, while the second stage refines the segmentation through a deeper network. It is trained end-to-end and ranked first in the BraTS 2019 competition, but the parameter volume also demands high hardware requirements.

Recent studies [14,15,16] demonstrate that transformers can significantly improve the efficiency of brain tumor segmentation. Inspired by the success of transformers in 2D visual tasks, Wang et al. [15] proposed the TransBTS based on the transformer. It captures global contextual information and achieves the best performance at the time. However, this model did not optimize the transformer, resulting in considerable computational complexity. To reduce complexity, Hatamizadeh et al. [16] introduced a sliding window attention mechanism based on the Swin-transformer [17], reducing model complexity. Although transformer-based segmentation methods have achieved notable performance, the quadratic complexity also increases model parameters and computational complexity. To address this, Lee et al. [18] replaced the attention module with a large kernel convolution module. While achieving comparable performance and reducing the parameters, it is merely a module replacement for the Swin-Transformer. It does not consider the local contextual information lost by large kernel convolution.

In summary, existing 3D methods demonstrate a superior ability to capture three-dimensional contextual information. However, certain limitations persist. Three-dimensional MRI images, composed of multiple slices in scans, often exhibit noise across different dimensions, necessitating networks with strong feature extraction capabilities and a keen sensitivity to local details. Contemporary mainstream segmentation models employ transformers as the backbone for feature extraction. While this approach can capture global information correlations, it fails to process local details finely. Secondly, the vast number of parameters inherent in current 3D segmentation models, often an order of magnitude greater than those in 2D models, imposes more stringent hardware requirements.

In response to these challenges, a lightweight MRI brain tumor segmentation method, Enhanced by Hierarchical Feature Fusion (EHFF), is proposed. This method effectively strengthens and merges features while reducing model parameters, achieving superior segmentation performance. The main contributions are as follows:

(1) An adaptive feature learning (AFL) module is constructed as part of a fine-grained feature adjustment network guided by global contextual information. By leveraging macro perception and micro focus, hierarchical features are strengthened, and model parameters are reduced.

(2) To address the lack of attention to local position details, a hierarchical feature weighting (HFW) module is designed. This module extracts refined features across multiple scales through multi-level weighted features, reinforcing the detailed features of spatial positions.

(3) To mitigate the easy loss of up-sampling features in the encoder–decoder structure, a hierarchical feature retention (HFR) module is constructed to retain, up-sample, and fuse features at each layer, thus achieving superior detail retention and reconstruction effects and acting as a supplementary decoder.

## 2. Proposed Method

**Figure 1:**
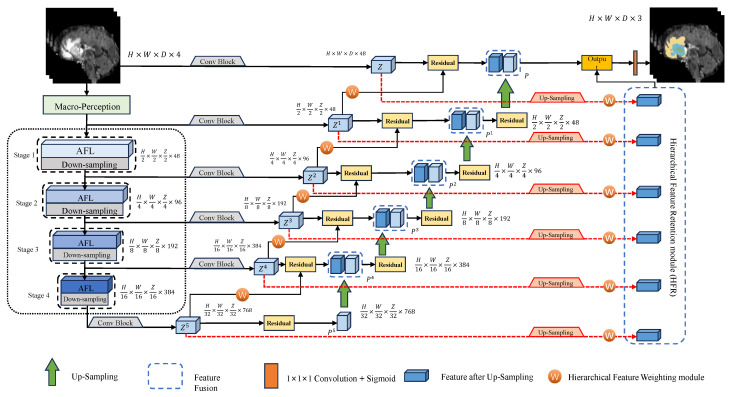
The overall architecture of EHHF.

The proposed EHFF is a lightweight brain tumor segmentation method that includes an encoder, a decoder, an HFW module, and an HFR module, as depicted in Figure 1. Initially, during the encoding phase, global contextual spatial information is captured through multilevel macro perception and micro focus to guide the enhancement of local fine-grained spatial features. Subsequently, the HFW module refines and weights each hierarchical feature through backpropagation, extracting multi-scale refined features to enhance the detailed features of spatial locations. Ultimately, as an auxiliary decoder, the HFR module collaborates with the primary decoder to perform up-sampling restoration on the refined features, collectively generating the anticipated segmentation image.

### 2.1. Encoder

**Figure 2:**
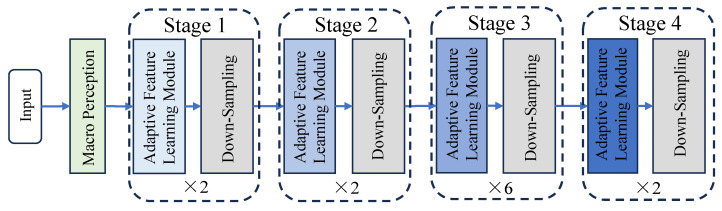
Multistage architecture of the encoder.

The encoder is designed to extract high-level features through a multi-level and multi-stage network, as illustrated in Figure 2. It captures the contextual information of brain tumor images and transmits them via skip connections to the decoder, thereby aiding the model in the segmentation results of the input MRI images.

For input 3D MRI brain tumor images, a macro-perception module is initially performed on them to generate a feature map with global contextual information, facilitating subsequent feature processing. The macro-perception module comprises several 7 × 7 × 7 large kernel convolutions and corresponding normalization layers. Compared to the mainstream Swin-Transformer-based method, this method of obtaining the global receptive field through large kernel convolutions occupies less memory and reduces model parameters notably. Particularly for the segmentation of MRI brain tumor images, the macro-perception module can preserve local detail features while processing the long-distance spatial position relationship of features.

**Figure 3:**
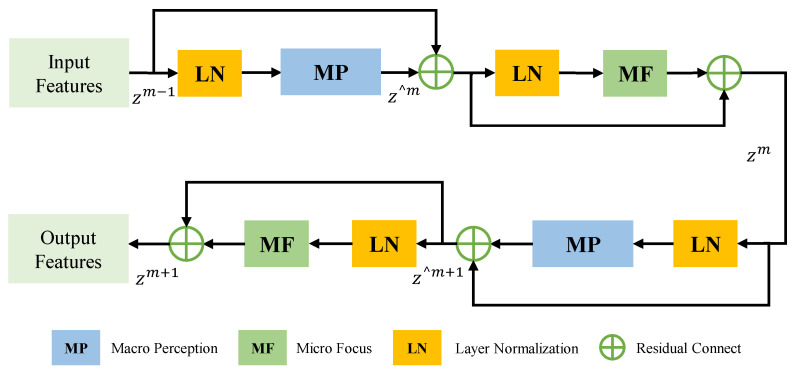
The flowchart of the AFL module.

Recent research indicates that the depth and complexity of the network often result in better performance [19,20]. A single macro-convolution module cannot fully capture the abstract features in complex data, nor can the original features of the MRI image be effectively transmitted. Therefore, a multi-stage deep encoding feature extraction network is designed. This encoding network includes four stages of cascaded AFL feature extraction and down-sampling modules, used to obtain the high-level feature gradually. The AFL module, shown in Figure 3, allows features to continuously learn more complex representation capabilities to better capture the abstract concepts and semantic information in the data and then better fit the data.

The AFL module comprises a macro-perception module and a micro-focus module. The macro-perception module is designed to capture the global features and extensive spatial contextual information of MRI brain tumor images. Given the substantial computational demands and parameters of 3D images, a single stack of macro-perception modules may lead to an explosion in model parameters, loss of local details, and compromised feature representation. Thus, inspired by the MLP layer in Swin-Transformer [17], a micro-focus module is constructed for fine-grained adjustments to enhance the expression and adaptability of spatial detail features. This approach compensates for the drawbacks of stacking large kernel convolutions, thereby improving the effectiveness. The 1 × 1 × 1 convolution in the micro-focus module further refines and boosts the abstract expression of features by introducing non-linear transformations [18], reducing parameter volume and computational complexity.

In the encoding phase, the macro-perception module initially maps the input MRI brain tumor images to acquire the preliminary feature map $z^{m-1}$. This preliminary feature map, with size H/2 × W/2 × D/2 and channel dimension 48, is fed into Stage 1 for further feature processing. To be efficient, the preliminary feature map is first normalized and then passed into the AFL module. The macro-perception module in the AFL module captures the deep global spatial contextual feature information to obtain the feature map $z^{\wedge m}$, and the preliminary feature map $z^{m-1}$ is fused through residual connection to gain the global spatial feature representation of different scales. However, relying on residual fusion results in feature redundancy, leading to rich feature information but a loss of attention to local spatial detail. Therefore, the micro-focus module is used to optimize $z^{\wedge m}$ to acquire the refined feature $z^{m}$, compressing each channel independently through 1 × 1 × 1 convolution, to decrease feature redundancy by minimizing cross-channel spatial context. This operation adjusts local spatial fine-grain to reduce model complexity, introducing non-linear transformations, enhancing the expressive ability, and enriching feature representation.

The above process is iterated twice in each stage, with the macro-perception module and the micro-focus module processing $z^{m}$ again. This approach optimizes the spatial detail features while obtaining the global receptive field and acquiring a more complete feature representation. Next, through a 2 × 2 × 2 size, with the stride of 2 down-sampling convolution block, the resolution of the feature map is reduced by a factor of 2, and the refined features are passed into the subsequent stage. Consequently, the output feature map size of Stage 1 is H/4 × W/4 × D/4 × 96. Stage 2–4 repeat the operations in Stage 1, and the sizes of the output feature maps are H/8 × W/8 × D/8 × 192, H/16 × W/16 × D/16 × 384, H/16 × W/16 × D/16 × 768, respectively.

The output features of these 4 stages are regarded as hierarchical features, which are applied to the subsequent HFW and HFR modules. They can obtain more comprehensive and rich multi-scale feature representation, acquiring the ability to perceive global spatial context information and local spatial details, which helps capture brain tumor features in MRI images. The above operations are expressed in the following formula:

(1) $$z^{\wedge m}=MP\left\{LN\left(z^{m-1}\right)\right\}+z^{m-1}$$

(2) $$z^{m}=MF\left\{LN\left(z^{\wedge m}\right)\right\}+z^{\wedge m}$$

(3) $$z^{\wedge m+1}=MP\left\{LN\left(z^{m}\right)\right\}+z^{m}\;$$

(4) $$z^{m+1}=MF\left\{LN\left(z^{\wedge m+1}\right)\right\}+z^{\wedge m+1}$$

where MP denotes the macro-perception module, MF denotes the micro-focus module, LN denotes the Layer Normalization layer, playing the role of regularization and optimization of training, $z^{m-1}$, $z^{m}$, and $z^{m+1}$, respectively, represent the input, the output after the first round of iterations, and the final output of the AFL module within each stage, $z^{\wedge m}$ and $z^{\wedge m+1}$ denote the outputs of the first and second MP blocks of the AFL module.

### 2.2. Decoder

The decoder is designed to progressively up-sample and fuse features of varying scales, originating from the outputs of stages 1 to 4 in the encoder. This process restores the resolution to that of the input image, ultimately generating the final segmentation result. The decoding process begins with up-sampling the highest-level features, which are output from Stage 4 of the encoder. The output features from the corresponding encoder stage are fused at each up-scaling layer via skip connections [21]. This fusion facilitates the capture of subtle brain tumor features and enhances the precision of brain tumor segmentation.

Specifically, feature maps of varying scales denoted as $Z^{1}$–$Z^{5}$, output by each encoder stage, are transformed by a convolution block. This block consists of two 3 × 3 × 3 convolution layers that intensify feature representation. The final encoding output of Stage 4, after a 3 × 3 × 3 convolution transformation, is labeled as $Z^{5}$ and serves as the input for the first layer of up-sampling. In the subsequent progressive up-sampling process, encoding feature information is introduced into the corresponding decoding stages, and the HFW module is used to apply weights to $Z^{1}$–$Z^{5}$. These weighted features are then connected to the next layer of the decoder via skip connections, enabling the acquisition of multi-scale refined features and improving feature accuracy. This assists further feature fusion and information transmission, thereby boosting model performance. In the terminal part of the decoding, segmentation output is computed through a 1 × 1 × 1 convolution layer and a sigmoid activation function, realizing pixel-level prediction of brain tumor segmentation. The above operations are expressed in the following formula:

(5) $$Z^{1}=Conv_{3\times 3}\left\{MP\left(input\right)\right\}$$

(6) $$Z^{i}=Conv_{3\times 3}\left(Stage_{i-1}\right),i\in \left[2,5\right]$$

(7) $$P^{5}=Up\left(Z^{5}\right)$$

(8) $$P^{i}=Up\left\{Z^{i},P^{i-1},HFW\left(Z^{i+1}\right)\right\},i\in \left[1,4\right]$$

(9) $$P=Up\{input,P^{1},HFW\left(Z^{1}\right)\}$$

where $Z^{1}$ denotes the features mapped by the first layer macro-perception module, $Conv_{3\times 3}$ denotes the Conv Block consisting of two 3 × 3 × 3 convolution kernels, $Z^{i}$ denotes the features mapped by the output of i-th Stage through Conv Block module, input denotes the input image, Up denotes the up-sampling, HFW denotes the hierarchical feature weighting module, $P^{i}$ denotes the output of the i-th up-sampling, and *P* denotes the final output of the decoder.

### 2.3. Hierarchical Feature Weighting Module

**Figure 4:**
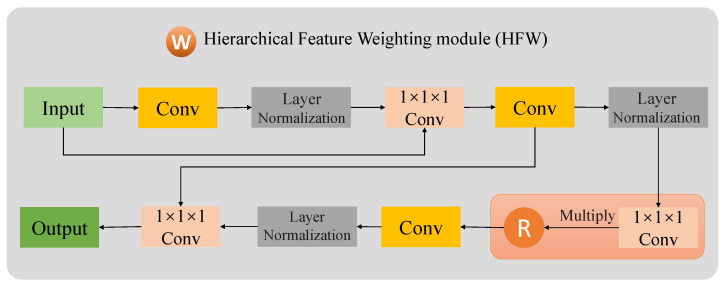
The flowchart of the HFW module.

The hierarchical feature weighting (HFW) module is designed to refine feature information through backpropagation, aiming to minimize information redundancy and enhance the feature representation, thereby augmenting model performance. Traditional encoder–decoder architectures typically obtain high-level features through down-sampling and pooling. However, a semantic gap often exists between the encoding and decoding processes, which hinders the integration of high-level and low-level features, resulting in blurred prediction boundaries. To address this issue, the HFW module was developed to retain hierarchical feature information from different encoder stages. By weighting features across various levels, the module facilitates a more effective fusion of high-level and low-level features. This approach reduces information loss during up-sampling and strengthens the precision of spatial features. The framework of the HFW module, as shown in Figure 4, incorporates a learnable weight parameter optimized through backpropagation. This optimization is based on the weighted learning of features $Z^{1}$–$Z^{5}$ from stages 1 to 4 through backpropagation, which determines the contribution of the output in each stage. This process extracts multi-scale refined features, enriching the detail of spatial locations.

Traditional methods of residual connection fusion, which involve simply adding or concatenating features, provide comprehensive feature representation but also introduce excessive feature redundancy and irrelevant information. To address this, a weighted fusion approach is employed, offering a more nuanced method of feature integration. This approach discards superfluous feature information, giving the model flexibility in information fusion and potentially reducing the risk of overfitting [22].

The HFW module takes the hierarchical features $Z^{1}$–$Z^{5}$, output by each stage of the encoder [23], as input, targeting the acquisition of local spatial details under a global receptive field. Initially, local spatial details of each hierarchical feature are captured through a convolutional block composed of multiple 3 × 3 × 3 convolutions and normalization layers. Subsequently, feature information is compressed and refined through several 1 × 1 × 1 and 3 × 3 × 3 convolutional kernels, yielding compressed refined features *E*. A learnable random variable *R* is then introduced, multiplied by the refined features *E*, and processed through another series of 3 × 3 × 3 convolutions, normalization layer, and 1 × 1 × 1 convolution to obtain the final weighted feature. Given the problem of information loss due to network depth, multiple residual connections are designed within the HFW module to ensure that the acquisition of refined local spatial detail features does not lead to gradient vanishing or feature loss. The above operations are expressed in the following formula:

(10) $$E=Conv_{1\times 1\times 1}{\left\{LN\left[Conv\left(input\right)\right]\right\}}_{2}$$

(11) $$Out=Conv_{1\times 1\times 1}\left\{LN\left[Conv\left(E\times R\right)\right]\right\}$$

where Conv denotes multiple 3 × 3 × 3 convolutional layer, $Conv_{1\times 1}$ denotes 1 × 1 × 1 convolutional layer, *R* denotes random parameter, *E* denotes compression refined feature, and subscript _2_ denotes that the operation loops twice.

### 2.4. Hierarchical Feature Retention Module

The hierarchical feature retention module is designed to function as a supplementary decoder, preserving hierarchical features $Z^{1}$–$Z^{5}$ and executing hierarchical up-sampling fusion. In the final stage, it collaborates with the primary decoder to decode and output the final predicted segmentation of brain tumor images. Contrary to the primary decoder, which integrates encoder and decoder features through skip connections, the HFR module focuses on the direct outputs of various stages. It directly performs hierarchical up-sampling on the preserved multi-scale encoding features, enabling the model to retain spatial information and relationships across different resolution feature maps. For 3D data of brain tumors, this spatial correspondence is crucial for capturing the connections between pixels and spatial details, significantly aiding in accurate measurement and analysis across multiple slices. This capability is instrumental in assisting the model to determine the volume and contour segmentation of brain tumors.

Regarding the retained information $Z^{1}$–$Z^{5}$ from the encoder, they are upscaled to produce five outputs with size H × W × D × 48, indirectly capturing different scale feature information at various spatial resolutions. Subsequently, these features are fused proportionally with the final output from the primary decoder to yield the ultimate segmentation prediction image. The above operations are expressed in the following formula:

(12) $$Q=\sum_{i=1}^{5}Up\left(Z^{i}\right)$$

(13) $$Output=Sigmoid\{Conv_{1\times 1\times 1}\left[P\times \partial +Q\times \left(1-\partial \right)\right]\}$$

where *Q* denotes the output of the HFR module, Up denotes up-sampling, Sigmoid denotes the activation function, and *∂* denotes the fusion coefficient, which is set to 0.8 according to the empirical value.

## 3. Experimental Results and Analysis

Experiments were conducted on the BraTS 2021 dataset [24], and the results were compared with other mainstream methods. The experiments were carried out using the PyTorch 1.10 deep learning framework in a Python 3.8 environment. The hardware setup consisted of a single 24GB NVIDIA GeForce RTX 3090. And the initial learning rate was set to 0.001. The model was trained for 300 epochs with a batch size of 1, utilizing the Adam optimizer for end-to-end optimization.

### 3.1. Dataset

**Figure 5:**
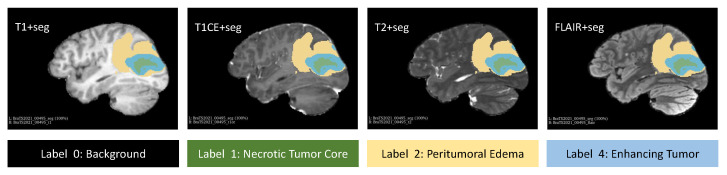
Visualization of 3D MRI images and brain tumor annotation.

The BraTS 2021 dataset comprises 1251 cases in the training set, 219 cases in the validation set, and 570 cases in the test set. Each MRI scan includes 3D images from four modalities. Within the training set, each case’s scan consists of 3D images from t1, t1ce, t2, flair modalities, and one shared label. The shared labels comprise four classification categories: 0, 1, 2, and 4. To gain a visual understanding of the brain tumor segmentation annotations and labels across different modalities of MRI images, a case was selected and visualized using the 3D Slicer medical image analysis software, as depicted in Figure 5. The final task of the BraTS 2021 Challenge necessitates the identification of three specific sub-regions: the enhanced tumor ET (label 4), the tumor core TC (labels 1 and 4), and the entire tumor WT (labels 1, 2, and 4). Since the organizers of this dataset did not make the test data publicly available, comparisons were made using a five-fold cross-validation method on the training data only.

### 3.2. Evaluation Metric

In terms of MRI brain tumor segmentation, the evaluation of segmentation performance commonly employs the Dice Similarity Coefficient (DSC) as the standard metric. The DSC serves as a widely used measure to quantify the similarity between segmentation results and reference standards. The formula is as follows:

(14) $$DSC=\frac{2|S\cap G|}{\left|S|+|G\right|}$$

where |S∩G| denotes the number of intersection pixels between the segmentation result and the standard label, while |S| and |G| represent the number of pixels in the segmentation result and the standard label, respectively. The DSC value ranges between 0 and 1, wherein a value closer to 1 indicates a higher degree of similarity between the segmentation result and the standard label.

### 3.3. Comparative Experiment

**Figure 6:**
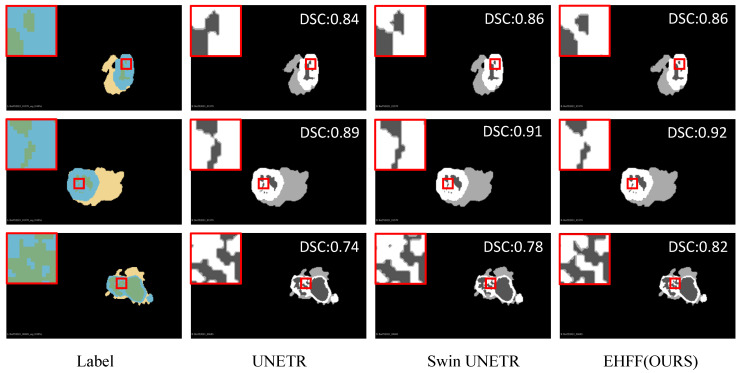
Segmentation comparison by various methods.

**Figure 7:**
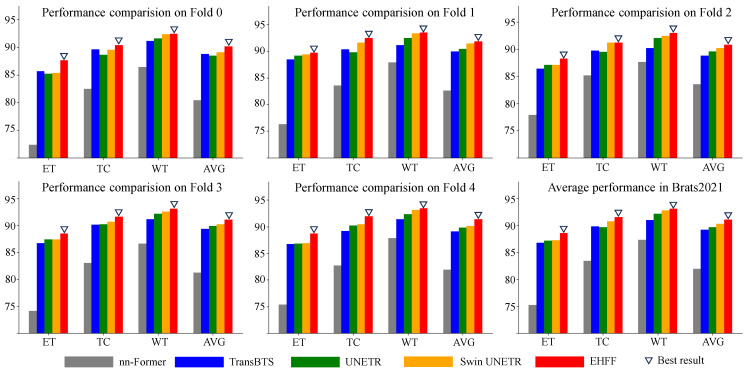
The visual comparison of the performance.

We conducted five-fold cross-validations using five subsets of the BraTS 2021 dataset. The proposed method was compared with other mainstream methods, including nn-Former [25], TransBTS [15], UNETR [14], and Swin UNETR [16], as illustrated in Table 1, Table 2, Table 3, Table 4 and Table 5. The visualization of these segmentation results is presented in Figure 6. Figure 7 presents a bar chart comparison, facilitating a more intuitive appreciation of the superiority inherent in our method. And the experimental results in Table 6 (including 3 additional methods: 3D-UX-Net [18], TransUNet [26], and MedNeXt-S [27]) revealed that, across three semantic categories (ET, TC, and WT) in five subsets, the DSC achieved by the proposed method surpassed those of competing methods, demonstrating considerable competitiveness. Specifically, the five-fold cross-validation results for ET, TC, and WT achieved by the proposed method were 88.57%, 91.53%, and 93.09%, respectively, with an average five-fold cross-validation result of 91.06%. This represents an average improvement of 0.83% of the BraTS 2021 dataset when compared to the advanced method, Swin UNETR. At the same time, the proposed method’s standard deviations for segmentation accuracy are 0.445, 0.477, and 0.139, respectively, which are at relatively low levels, indicating our stability.

Further, Table 7 provided a comparison of the parameter number and inference time across different methods. It is evident that the parameter count and the inference time for the proposed method are significantly lower than that of other methods, with a reduction of approximately 29% compared to Swin UNETR. This demonstrates the relative simplicity of the proposed method and further substantiates the lightweight nature of the EHFF.

Compared to methods like nn-Former, UNETR, Swin UNETR, and TransUNet, which use the transformer for global context modeling, our method offers significant advantages. The high computational complexity of the transformer leads to large model parameters and inefficiencies, especially when handling long sequences. In contrast, our proposed method uses a macro-perception module built with large kernel convolutions to model global context, significantly reducing the number of model parameters. Combined with a micro-focus module, EHFF effectively captures image features. And the HFW module is employed to refine these features, enabling precise brain tumor segmentation.

Although our method does not have fewer parameters than TransBTS, it performs better overall. In the five-fold cross-validation on the BraTS 2021 dataset, EHFF surpassed TransBTS in all 15 metrics, especially achieving nearly 2% higher accuracy in the WT index. This distinction highlights the superior effectiveness of our method, even with a slightly larger parameter count.

When compared to 3D UX-NET, both methods abandon the transformer. However, 3D UX-NET focuses too much on global features, neglecting the finer details of brain tumors. Our method improves on this by preserving important detail information through hierarchical feature weighting, ensuring precise segmentation by enhancing attention to tumor details across multiple scales.

Compared to the latest method, MedNeXt-S, our method has certain limitations. MedNeXt-S makes deeper use of CNNs, extracting higher-level information through deeper convolutional modules. This further enhances the representation of tumor details, improving segmentation performance. This design has inspired us, and in future research, we plan to explore deeper CNN architectures to achieve even better performance while maintaining or reducing model complexity.

### 3.4. Ablation Experiment

To validate the efficacy of the modules within the proposed EHFF, ablation experiments were conducted on two key components: the HFW module and the HFR module. In order to efficiently showcase the effectiveness of these modules, the experiments were exclusively performed on a subset of the BraTS 2021 dataset, specifically fold 4. The experimental results clearly demonstrate that the EHFF achieves its optimal performance when both the HFW module and the HFR module are employed together in Table 8a. In addition, ablation experiments were carried out on the fusion coefficient, as indicated in Table 8b. The results reveal that the optimal performance is achieved when the fusion coefficient is set to 0.8.

### 3.5. Limitations

Brain tumor segmentation is a task that involves multi-scale feature processing. The proposed method uses large kernel convolutions to extract global features. However, the use of large kernel convolutions can reduce the ability to capture fine details. In this study, we enhanced tumor detail features through a micro-focus module. Despite this, the method is still not detailed enough and may result in the loss of some tumor details. This issue is also common in other current brain tumor segmentation methods. In the future, we plan to design a more refined local feature processing module to work alongside large kernel convolutions, aiming to achieve a more lightweight and precise brain tumor segmentation.

## 4. Conclusions

In this paper, we propose a lightweight MRI brain tumor segmentation method enhanced by hierarchical feature fusion to achieve precise segmentation of MRI brain tumors. Initially, the AFL module is constructed to thoroughly capture the global features of MRI brain tumor images by embracing macro perception and micro focus. This module subsequently refines these features at a granular level, thereby reducing computational complexity while enhancing the spatial information. Secondly, the HFW module is developed to extract and augment multi-scale features through multi-level weighted processing. This module can enhance the detail features of spatial locations and mitigate the shortfall in local detail attention. Finally, hierarchical features are fused and up-sampled through the HFR module, which co-predicts the accurately segmented brain tumor images in conjunction with the decoder. The five-fold cross-validation results on the BraTS 2021 dataset show that the proposed method achieves standard deviations of 0.445, 0.477, and 0.139 in the ET, TC, and WT categories, respectively. These low standard deviations demonstrate the stability of the proposed method. Additionally, the average segmentation accuracy of the proposed method, measured by DSC, reaches 88.27%, 91.31%, and 92.96% for these categories, which are 1.34%, 0.83%, and 0.34% higher than those of Swin UNETR, respectively. These results demonstrate that the proposed method can effectively improve the quality of segmented images and provide better clinical support for brain tumor segmentation imaging.

In general, while our method demonstrates obvious advantages over others, it still faces limitations in practical clinical applications. For instance, deep learning-based brain tumor segmentation requires significant computational resources and costly annotated datasets, which increases the overall cost. Looking ahead, our future work will involve using cloud computing and distributed learning to reduce computational costs, thereby achieving more lightweight brain tumor segmentation.

## Figures and Tables

**Table 1:** Performance comparison on Fold 0 in the BraTS 2021 dataset.

	nn-Former	TransBTS	UNETR	Swin UNETR	EHFF
ET	72.34	85.67	85.23	85.33	**87.63**
TC	82.46	89.64	88.64	89.54	**90.39**
WT	86.45	91.12	91.61	92.32	**92.45**
Avg	80.42	88.81	88.49	89.06	**90.16**

Note: the bold denotes the best result.

**Table 2:** Performance comparison on Fold 1 in the BraTS 2021 dataset.

	nn-Former	TransBTS	UNETR	Swin UNETR	EHFF
ET	76.31	88.41	89.13	89.41	**89.68**
TC	83.54	90.32	89.77	91.64	**92.45**
WT	87.88	91.10	92.49	93.31	**93.47**
Avg	82.58	89.94	90.46	91.45	**91.87**

**Table 3:** Performance comparison on Fold 2 in the BraTS 2021 dataset.

	nn-Former	TransBTS	UNETR	Swin UNETR	EHFF
ET	77.91	86.41	87.11	87.12	**88.29**
TC	85.15	89.75	89.54	91.21	**91.24**
WT	87.65	90.24	92.12	92.45	**92.98**
Avg	83.57	88.80	89.59	90.26	**90.84**

**Table 4:** Performance comparison on Fold 3 in the BraTS 2021 dataset.

	nn-Former	TransBTS	UNETR	Swin UNETR	EHFF
ET	74.12	86.74	87.39	87.42	**88.52**
TC	83.02	90.11	90.23	90.69	**91.64**
WT	86.63	91.13	92.14	92.54	**93.12**
Avg	81.26	89.33	89.92	90.22	**91.09**

**Table 5:** Performance comparison on Fold 4 in the BraTS 2021 dataset.

	nn-Former	TransBTS	UNETR	Swin UNETR	EHFF
ET	75.36	86.72	86.78	86.87	**88.74**
TC	82.71	89.21	90.24	90.44	**91.91**
WT	87.82	91.41	92.36	93.12	**93.45**
Avg	81.96	89.11	89.79	90.14	**91.37**

**Table 6:** Average DSC performance comparison on Fold 0-4 in Brats2021 dataset.

	nn-Former	TransBTS	UNETR	Swin UNETR	3D-UX-Net *	TransUNet *	MedNeXt-S *	EHFF	Std
ET	75.21	86.79	87.13	87.23	-	-	-	**88.57**	0.445
TC	83.38	89.81	89.68	90.70	-	-	-	**91.53**	0.477
WT	87.29	91.00	92.14	92.75	-	-	-	**93.09**	0.139
Avg	81.96	89.20	89.65	90.23	90.63	89.17	**91.27**	90.85	0.361

Note: - indicates missing data, * indicates data obtained directly from the original paper, and the Std denotes the standard deviation of the proposed EHFF.

**Table 7:** Parameters and inference time comparison.

Method	Parameters	Inference Time (s)
TransBTS	**32,987,588**	-
nn-Former	149,478,267	-
UNETR	125,018,259	-
Swin UNETR	62,187,296	0.96
**EHFF (Ours)**	44,053,394	**0.93**

**Table 8:** Abalation experiments.

(a) Module Abalation Comparison.			(b) Fusion Coefficient.	
**HFW**	**HFR**	**DSC**	**Fusion Coefficient**	**DSC**
×	×	89.87	0.6	90.88
✓	×	90.63	0.7	91.18
×	✓	90.74	0.8	**91.37**
✓	✓	**91.37**	0.9	90.77

## Data Availability

Data are contained within the article.
